# Fracture resistance of monolithic translucent zirconia crown bonded with different self-adhesive resin cement: influence of MDP-containing zirconia primer after aging

**DOI:** 10.1186/s12903-023-03365-5

**Published:** 2023-09-05

**Authors:** Shaima Tyor, Walid Al-Zordk, Amal Abdelsamad Sakrana

**Affiliations:** 1 Department of Fixed Prosthodontics, Faculty of Dentistry, Mansoura University, Mansoura, Egypt; 2 Department of Fixed Prosthodontics, Faculty of Dentistry, Horus University, New Damietta, Egypt

**Keywords:** Zirconia primer, Single-step adhesive, Resin cements, Translucent zirconia, Fracture resistance

## Abstract

**Objectives:**

Successful ceramic restorations depend on the strong bonding with resin cement and even stress distribution. The aim of this study was to evaluate the impact of adding MDP-containing zirconia primer before self-adhesive resin cements with different functional acidic monomers on fracture resistance of monolithic zirconia crown.

**Materials and methods:**

Eighty defect-free human maxillary premolars were divided according to the cement type and application of MDP-containing zirconia primer into eight groups (n = 10): Calibra Universal (C), Calibra Universal combined with zirconia primer (CZ), RelyX U200 (R), RelyXU200 combined with zirconia primer (RZ), Panavia SA Cement Plus (P), Panavia SA Cement Plus combined with zirconia primer (PZ), Multilink Speed (M), and Multilink Speed combined with zirconia primer (MZ). After teeth preparation and fabrication of zirconia crowns, each crown was bonded to its corresponding tooth. All specimens were subjected to 10,000 thermocycles between 5 and 55°C, followed by cyclic load (50 N) for 240,000 cycles. Each specimen was subjected to a static axial load until fracture using universal testing machine and the fracture load was recorded. The fracture mode studied and recorded. The fracture load results were analyzed using two-way ANOVA test (α = 0.05).

**Results:**

A significant interaction (P = 0.038) of combining MDP-containing zirconia primer and cement type on fracture resistance of monolithic zirconia crown was detected. The mean fracture load values of zirconia crown were significantly influenced by the combined application of the MDP-containing zirconia primer with Calibra Universal (P = 0.01), RelyX U200 (P < 0.001), and Multilink Speed (P = 0.038), while there was no significant difference with Panavia SA Cement Plus (P = 0.660). There was significant difference (F = 20.69, P < 0.001) between the mean fracture loads of groups with self-adhesive cements (C, R, P, and M groups). The highest fracture load was recorded with RZ group (2446.90 ± 126.72 N) while the lowest fracture load was recorded with C group (1623.18 ± 149.86 N).

**Conclusions:**

The self-adhesive resin cement with different acidic functional monomer affects the fracture resistance of monolithic zirconia crown. Application of MDP-containing primer could improve the fracture resistance of monolithic zirconia crown with most self-adhesive cements. The application of an MDP-containing primer had no impact on the fracture resistance of monolithic translucent zirconia crown bonded by MDP-containing self-adhesive resin cement.

## Introduction

The clinical application of zirconia restoration has gained popularity because of its mechanical properties and biocompatibility [1]. However, one of the drawbacks of the conventional zirconia was its limited translucency [2]. The microstructure of subsequent generation of zirconia was refined so that its grain boundaries do not interfere with light [3]. Furthermore, the new generations of zirconia have been obtained with enhanced optical characteristics by introducing larger amounts of cubic phase [4]. Despite the enhanced translucency of recent zirconia, its high cubic content markedly decreased its strength [5].

Adhesion between the restoration and tooth is critical for successful clinical performance of indirect restoration [6–8]. Unlike glass-ceramics, zirconia is acid-resistant material because of its glass-free polycrystalline microstructure [9–12]. Both mechanical and chemical pre-treatments are recommended for zirconia bonding [13, 14]. It was reported that, air-borne particle abrasion combined with application of MPD-containing primer improved the bonding between zirconia and resin cement [15–20].

Self-adhesive resin cements were designed to adhere to tooth structure in one-step protocol without the steps of etching, rinsing as well as priming [21]. Self-adhesive resin cements are clinically attractive due to their one step application and ease of usage even though the luting procedure is technique sensitive [22]. Any self-adhesive resin cement composed mainly of the predominant functional acidic monomers and conventional di-methacrylate monomers (such as Bisphenol A glycidyl methacrylate, Urethane dimethacrylate, and triethyleneglycol dimethacrylate) [23, 24]. The functional acidic monomers commonly used in self-adhesive resin cements are bis 2-methacryloxyethyl acid phosphate (BMP), 10-methacryloyloxydecyl dihydrogen phosphate (MDP), 4-methacryloxyethyl trimellitic anhydride (4-META), pyromellitic glycerol dimetracrylate (PMGDM), 2-methacryl-oxyethyl phenyl hydrogen phosphate (Phenyl-P), and dipentaerythritol penta-acrylate monophosphate (Penta-P) [22, 25, 26]. The initial low pH and high hydrophilicity of the self-adhesive resin cements promotes surface demineralization similar to what occurs with self-etching adhesives [27]. The predominant functional acidic monomers could chemically interact with zirconia and hydroxyapatite in tooth structure [11, 28–30].

The fracture strength of a ceramic restoration is influenced by a number of factors such as elastic modulus of the supporting substrate, loading force, and cementation procedures [31–34]. It was shown that the cement type affect the distribution of the stresses generated on the tooth-restoration complex and help to dissipate the occlusal forces applied to the restoration away from the tooth-restoration interface [35–37]. Weak bonding between the ceramic restoration and the resin cement results in uneven stress distribution and increased failure susceptibility [38]. The fracture of ceramic restoration can originate at the intaglio surface or the cementation interface at which the tensile stresses are concentrated [32].

When selecting a self-adhesive resin cement, the addition of MDP-containing primer to enhance the performance of zirconia crown need to be studied. Thus, the aim of this study was to evaluate the fracture resistance of monolithic translucent zirconia crown bonded by using self-adhesive resin cements with different functional acidic monomers. Also, the effect of combining MDP-containing zirconia primer with the self-adhesive resin cements with different functional acidic monomers on fracture resistance of monolithic translucent zirconia crown was studied. The first null hypothesis was that the type of self-adhesive resin cement would not affect the fracture resistance of monolithic translucent zirconia crown. The second null hypothesis was that the MDP-containing zirconia primer combination with self-adhesive resin cement with different functional acidic monomers would not affect the fracture resistance of monolithic translucent zirconia crown.

## Materials and methods

The materials used in this study are presented in Table 1. A total of 80 human maxillary first premolars extracted for orthodontics purposes were collected for this study. The sample size was calculated based on a previous study using G*power version (3.0.10) where α = 0.05 and 80.0% power [39]. The selected teeth were debrided and examined to be free from any stains, calculus and cracks. The average dimensions of selected teeth were 4.5 ± 0.5 mm in occluso-cervical direction, 7.3 ± 0.5 mm in mesio-distal direction, and 9 ± 0.5 mm from bucco-palatal direction. This study was approved by the Ethical Committee, Faculty of Dentistry, Mansoura University (Code: A05100221). To avoid dehydration, all teeth were stored in distilled water at room temperature through all testing period [40].

**Table 1:** The materials used in the study

Material	Product name	Patch number	Composition	Manufacturer
Zirconia	Katana Zirconia HTML (A2)	ECLCN	Mainly ZrO_2_ and 5%mol Y_2_O_3_	Kuarary Noritake Dental, Japan
Self-adhesive cement	Calibra Universal (Translucent)	00074644	Base: UDMA, Polymerizable trimethacrylate resin, Polymerizable dimethacrylate resin. Catalyst: UDMA, Urethane Modified Bis-GMA dimethacrylate resin, Polymerizable dimethacrylate resins, PENTA	Dentsply Sirona, Germany
	RelyX U200 (TR)	7,847,918	Base: Silane treated glass powder, 2-propenoic acid, 2-methyl-,1,1’-[1-(hydroxymethyl)-1,2-ethanediyl] ester, reaction products wıth 2 hydroxy-1,3-propanediyl dimethacrylate and phosphorus oxide, TEGDMA, silane treated silica, sodium persulfate, glass powder, tert-butyl peroxy-3,5,5-trımethylhexanoate. Catalyst: Silane treated glass powder, substituted dimethacrylate1-benzyl-5-phenyl-barbıc-acid, calcium salt, silane treated silica, sodium p-toluenesulfinate, 1,12-dodecane dimethycrylate, calcium hydroxide, methacrylated aliphatic amine, titanium dioxide	3 M ESPE, Neuss, Germany
	Panavia SA Cement Plus (Translucent)	3S0232	Base: BisGMA, TEGDMA, UDMA, 10-MDP, silanized glass filler, silanized colloidal silica, photo-initiator, chemical-initiator. Catalyst: Bis-GMA, dimethacrylate, silanized Barium glass filler, silanized colloidal silica, chemical accelerator, pigment.	Kuarary Noritake Dental, Japan
	Multilink Speed (Transparent)	Z015TF	Base: UDMA, TEGDMA, PEGDMA. Catalyst: ytterbium trifluoride, UDMA, TEGDMA, methacrylated phosphoric acid, PEGDMA	Ivoclar Vivadent, Schaan, Liechtenstein
Zirconia primer	Clearfil Ceramic Primer Plus		3-MPS, 10-MDP, Ethanol	Kuarary Noritake Dental, Japan

ZrO_2_: Zirconium dioxide, Y_2_O_3_: Yttrium oxide, BisGMA: Bisphenol A glycidyl methacrylate, TEGDMA: Triethyleneglycol dimethacrylate, UDMA: Urethane dimethacrylate, PEGDMA: Polyethylene glycol dimethacrylate,10-MDP: 10-methacryloyloxydecyl dihydrogen phosphate, PENTA: Dipentaerythritol Penta-acrylate Phosphate, 3-MPS: 3-methacryloxypropyltrimethoxysilane

According to the cement type and application of ceramic primer, the teeth were randomly divided into eight groups (n = 10): Calibra Universal (C), Calibra Universal combined with zirconia primer (CZ), RelyX U200 (R), RelyXU200 combined with zirconia primer (RZ), Panavia SA Cement Plus (P), Panavia SA Cement Plus combined with zirconia primer (PZ), Multilink Speed (M) and Multilink Speed combined with zirconia primer (MZ). Panavia SA Cement Plus was the only self-adhesive resin cement which contain MDP in its composition.

The teeth were marked 2 mm away from the cemento-enamel junction using permanent marker (0.4 mm OH Pen universal, Stabilo, Germany). For simulation of the periodontal ligaments, the root of each tooth was dipped into the molten wax at 2 mm away from the cement-enamel junction and left to be hardened. The root of each tooth was embedded vertically within acrylic resin (Cold cure acrylic material, Acrostone, Egypt). Then, the tooth was removed from the acrylic resin blocks leaving an alveolus-like acrylic mold. The roots and the acrylic mold were cleaned carefully by hot water to remove any wax remnant. Then, the root and its acrylic mold were painted with an adhesive (Identium Adhesive, Kettenbach, Germany) and left to dry for 5 min [41]. A poly Vinyl Siloxane light body (Ghenesyl light body, LASCOD, Italy) was injected into the acrylic resin mold and the teeth were re-inserted into the acrylic resin blocks and pressed to the same position to simulate the periodontal ligament [42]. A pre-preparation silicon index (Ghenesyl putty soft, LASCOD, Italy) was fabricated.

**Fig. 1:**
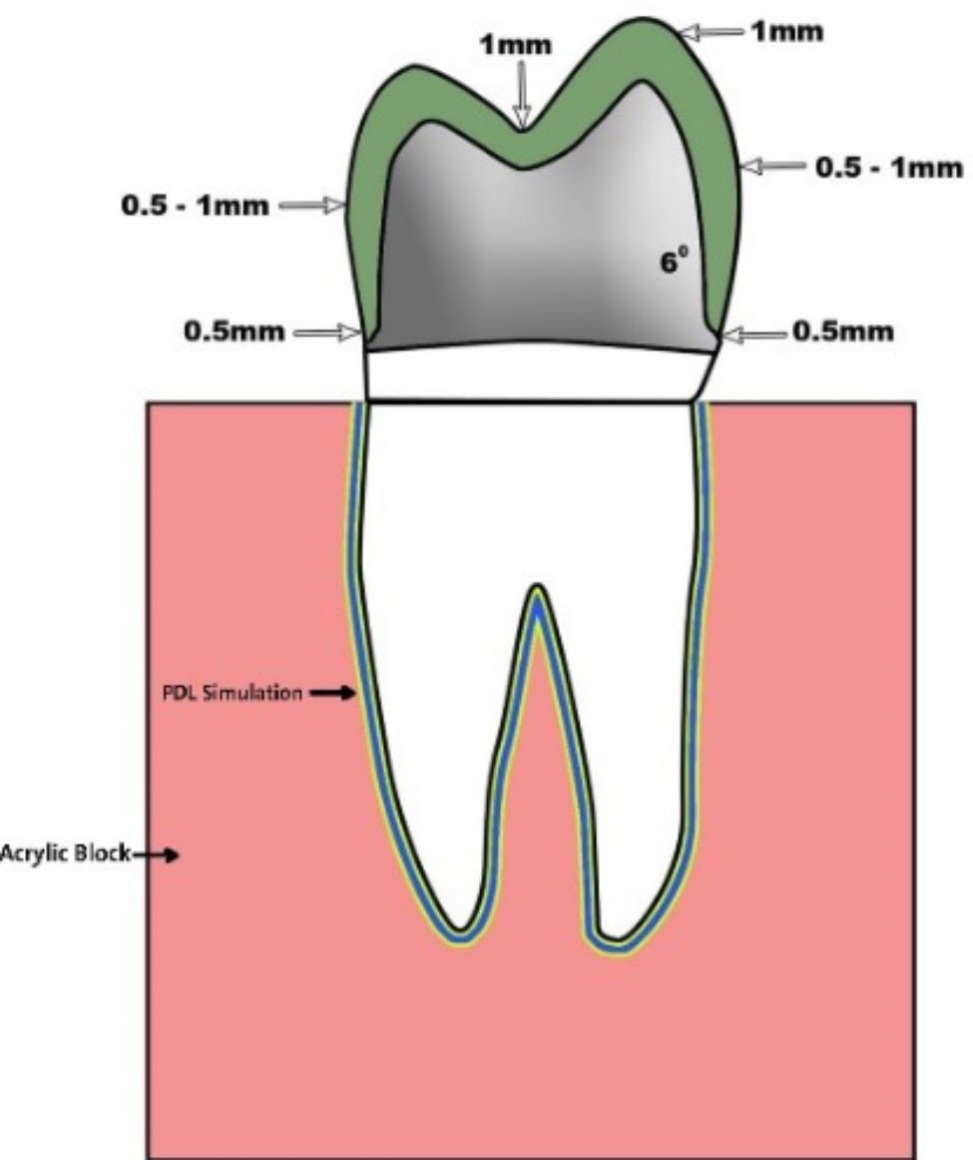
Illustration showing the tooth preparation

The teeth were prepared, using a dental surveyor (Marathon-103 surveyor, Saeyang Co., Korea), with the following parameters: 6-degree taper, 1 mm reduction for the non-functional cusp, 1.5 mm reduction for the functional cusp, and 0.5 mm chamfer finish line (Fig. 1). The occlusal preparation was performed using high speed handpiece (NSK-Nakanishi International, Japan) with tapered round end diamond stone supplied with air water coolant. While the axial preparation was performed using a dental surveyor attached to low speed straight hand piece supplied with external water coolant. The preparation was started with black-coded, 6-degree taper round end diamond stone (TR-12, MANI, Tochigi, Japan). The same previously used stone, size and taper, red-coded round end diamond stone followed by yellow-coded stone were used for finishing the preparation. The tooth preparation was checked with aid of a preparation putty index. All preparation was performed by single operator.

Fabrication of zirconia crowns: Each tooth was scanned using an optical scanner (Identica hyprid, MEDIT corp, Korea), then the software (Dental DB 2.2 Valletta, exocad GmbH, Darmstadt, Germany) created a virtual model from the scanning data. A virtual custom tooth model (alternative model) of a maxillary first premolar tooth was selected from the software library to be used with minimal modification to meet the standard design. The thicknesses of the designed restorations were checked using the measurement tool and the cut section view. Each design file was submitted to the milling machine (CORiTEC 250i touch, imes-icore, Germany) for milling of the crowns from a zirconia block (Katana HTML, Kuraray Noritake Dental Inc., Japan). Then, the milled crowns were sintered (conventional sintering program) using a sintering furnace (Tabeo-1/M/Zirkon-100, MIHM-VOGT, Germany) at 1500^°^C according to the instructions of the manufacturer. Finally, a thin glaze layer (CERABIEN ZR FC, Kuraray Noritake Dental Inc., Japan) was applied to the crowns and subjected to glaze firing using a porcelain furnace (Multimat Cube press, Dentsply Sirona, Germany) furnace. The intaglio surface of zirconia crown was air-borne particle abraded using 50 μm alumina particles at 2 bar air pressure for 10 s from a distance of 10 mm [41]. After that, the crowns were cleaned in ultrasonic cleaner (Codyson CD-4820, Shenzhen Codyson Electrical Co., Ltd, China) for 5 min then, dried for 10 min. Cementation: For CZ, RZ, PZ, and MZ groups, the intaglio surface of the crown was rubbed carefully by MDP-containing zirconia primer (CLEARFIL Ceramic Primer Plus; Kuraray Noritake Dental Inc., Japan) for 60 s using small micro-brush. For all groups, the corresponding self-adhesive resin cement was used for cementation of its corresponding tooth. The cement paste was dispensed on intaglio surface of the crown. Subsequently, the crown was carefully seated on its corresponding prepared tooth and kept under a constant static pressure of 10 N [43].

The crown was subjected to tack-curing (Elipar Deep Cure-S, 3 M ESPE Dental, St. Paul, MN, USA) according to the manufacturer’s instructions and the excess cement was removed using a scalpel. Then, each surface was completely cured according manufacture instructions with light intensity (1,470 mW/cm^2^) 20 s per surface for final curing except Panavia SA Cement Plus 10 s per surface as instructed. The specimen was kept isolated for 5 min to allow for the chemical curing. The margin was finished using a finishing stone (TR-25EF, 18,110,104, MANI, INC, Japan) and polished using an impregnated diamond polisher (DIACOMP PLUS RA - DCP-w11m, EVE, Germany). The cemented specimens were kept in distilled water at 37℃ for 24 h.

Aging: The specimens were subjected to 10,000 thermal cycles (Thermo Scientific, ThermoFisher Scientific Inc., Waltham, MA, USA) with 30 s dwell time in each water bath (5 and 55°C) and 5 s transition time. Using a chewing simulation unit (Chewing Simulator CS-4.4; SD Mechatronik), specimens were subjected to 240,000 cycles (frequency = 1.6 Hz) to simulate a unidirectional vertical force of 50 N [43]. The load was applied vertically parallel to the long axes of the teeth with a 6 mm ball-shaped antagonist in the center of the occlusal surfaces contacting the buccal and palatal cusps of each specimen [40].

Fracture resistance test: all specimens were subjected to load-to-fracture with compressive load using a universal testing machine (3345, Instron, USA). The load was applied with a 5 mm stainless steel ball perpendicular to the occlusal surface with a crosshead speed of 0.5 mm/min. The fracture load of each specimen was recorded in Newton (N). The fracture mode of each specimen was examined using a stereomicroscope (SZ61TR, Model SZ2-ILST, Olympus Co., Japan) at a magnification of x20. Fracture mode was classified into: minimal fracture or crack in the crown (Class I), less than half of the crown lost (Class II), half of the crown lost (Class III), more than half of the crown lost (Class IV), and sever fracture of the crown and/or the tooth (Class V) [43]. Classes I, II, III and IV were classified as non-catastrophic fracture, while class V was considered as catastrophic fracture. Representative specimens were selected for examination using the Scanning Electron Microscopy (JSM.6510LV, JEOL Ltd., Japan) at a magnification of x14 and x20. Before examination, the fracture surfaces of the specimens were coated with a 10 nm layer of gold using a sputter coating evaporator (SPI Module-Sputter Carbon/GoldCoater, SPI Supplies, USA).

Statistical analysis: data were statistically analyzed using statistical software (SPSS Version 22.0, IBM Corp, Armonk, NY). The Shapiro-Wilk test was used to confirm the normality of data. The fracture load results were analyzed using two-way ANOVA test was performed to determine the effect of MDP-containing zirconia primer combination with self-adhesive resin cement. Post Hoc Tukey test was used for multiple comparisons. Chi-Square test was applied to compare the fracture modes. Significance of the obtained results was judged at α = 0.05.

## Results

The combined interaction effect of the zirconia primer and cement type had a significant impact (P = 0.038) on the fracture resistance of the zirconia crown, according to the results of the two-way ANOVA test (Table 2). While Table 3 displays the mean fracture load values for the examined groups. The mean fracture load values of zirconia crown were significantly influenced by the combined application of the MDP-containing zirconia primer with Calibra Universal (P = 0.01), RelyX U200 (P < 0.001), and Multilink Speed (P = 0.038), while there was no significant difference with Panavia SA Cement Plus (P = 0.660). One-way ANOVA test revealed significant difference (F = 20.69, P < 0.001) between the mean fracture loads of groups with self-adhesive cements (C, R, P, and M groups). The mean fracture load value of group C was significantly lower than group R (P < 0.001), group M (P < 0.001), and group P (P < 0.001). The highest fracture load was recorded with RZ group (2446.90 ± 126.72 N) while the lowest fracture load was recorded with C group (1623.18 ± 149.86 N).

**Table 2:** Two-way ANOVA test for interaction effect between cement and primer̸cement variables on fracture resistance of monolithic zirconia restoration

Source	Type III Sum of Squares	df	Mean Square	F	*P*
Corrected Model	4.463E6^a^	7	637616.383	26.013	0.001
Intercept	3.416E8	1	3.416E8	13937.083	0.001
Primer	590442.248	1	590442.248	24.089	0.001
Cement	3652688.423	3	1217562.808	49.674	0.001
Primer * Cement	220184.011	3	73394.670	2.994	0.036
Error	1764802.910	72	24511.152	-	-
Total	3.478E8	80	-	-	-
Corrected Total	6228117.592	79	-	-	-

a. R Squared = 0.717 (Adjusted R Squared = 0.689)

**Table 3:** The mean and standard deviation (± SD) of fracture resistance values (N) of study groups

	Without primer	With primer	*P*
Calibra Universal	1623.18 ± 149.86^a^	1803.01 ± 133.18^c^	0.01
RelyX U200	2119.94 ± 205.93^b^	2446.90 ± 126.72^d^	< 0.001
Panavia SA Cement Plus	2091.72 ± 144.98^b^	2124.54 ± 181.59^d^	0.660
Multilink Speed	2087.28 ± 155.64^b^	2234.95 ± 138.59^d^	0.038

Different superscript letters in the same column indicate significant difference

**Fig. 2:**
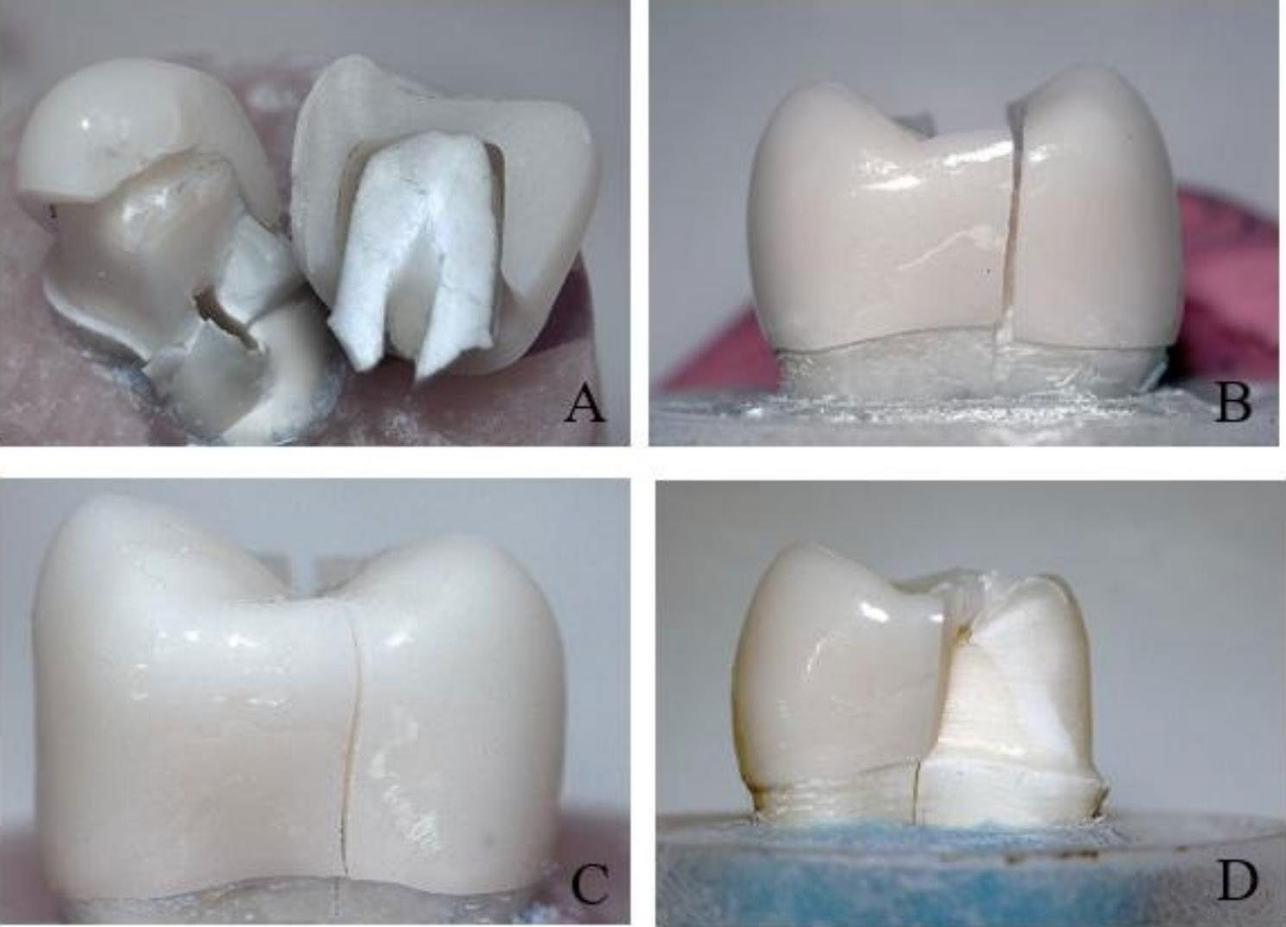
Stereomicroscopic images showing catastrophic fracture of monolithic zirconia crowns cemented with different cements. **A** Calibra Universal cement. **B** RelyX U200 cement. **C** Panavia SA Cement Plus. **D** Multlink Speed cement

**Fig. 3:**
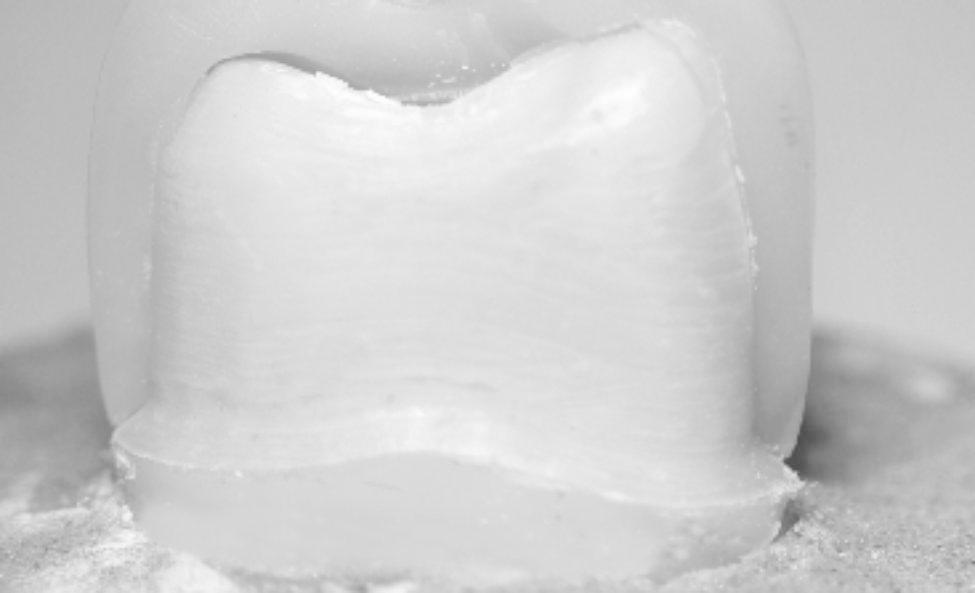
Stereomicroscopic image showing the fractured specimen with cement attached to the tooth

**Fig. 4:**
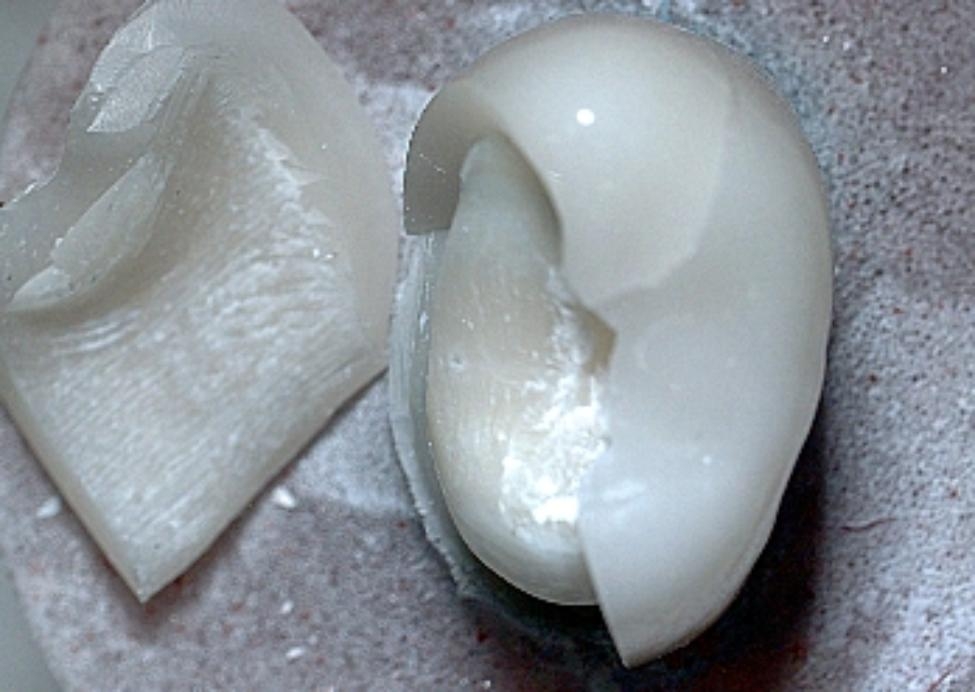
Stereomicroscopic image showing the fractured specimen with cement remnant on the intaglio surface of zirconia fragment

Chi-Square test (Table 4) showed that there was a statistically significant difference with higher catastrophic fracture mode among groups without MDP-containing zirconia primer (Fig. 2) than with MDP-containing zirconia primer combination groups. Also, Stereomicroscopic examination of fractured specimens showed that cement attached to tooth structure (Fig. 3) in without primer groups (C, R, P, and M), while cement remnants attached on the intaglio surfaces of zirconia crown (Fig. 4) were observed in primer̸cement groups (CZ, RZ, PZ, and MZ).

**Table 4:** Comparison of failure modes between studied groups

Groups	Non-catastrophic failure	Catastrophic failure	P
Calibra Universal	7(70)	3(30)	0.263
Calibra Universal with Primer	9(90)	1(10)	
RelyX U200	5(50)	5(50)	0.159
RelyX U200 with Primer	8(80)	2(20)	
Panavia SA Cement Plus	6(60)	4(40)	0.121
Panavia SA Cement Plus with Primer	9(90)	1(10)	
Multilink Speed	7(70)	3(30)	0.05
Multilink Speed with Primer	10(100)	0(0.0)	

**Fig. 5:**
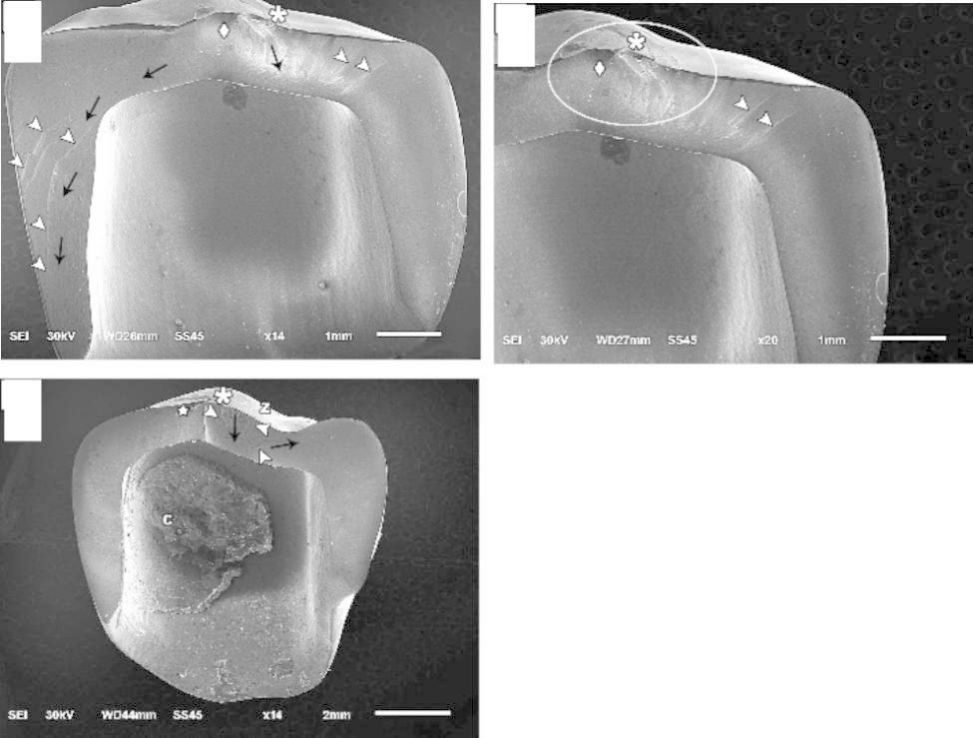
Representative SEM images of fractured zirconia fragments bonded by self-adhesive cement. (**A** and **B**) without primer. **C** with MDP-containing zirconia primer combined with self-adhesive resin cement Asterisk: Indicates the origin of fracture has the highest tensile stress level which was at the occlusal loading area surrounded by rougher area called fracture mist (star) Arrow head: Refers to hackle lines indicating the direction of crack propagation (black arrow) Diamond: Compression curls which is a curved lip immediately before the fracture changes and its direction before the final fracture occurs C: Cement

SEM analysis showed that the main origin of the fracture was detected at the occlusal surface from the main contact loading area. Few specimens had secondary origins near the major ones at the occlusal surface. The hackle lines, which represent the path and direction of crack propagation, were directed in corono-apical direction in all fractured specimens (Fig. 5).

## Discussion

The first null hypothesis, that the type of self-adhesive resin cement would not affect the fracture resistance of monolithic zirconia crown, was rejected because the results of the present study revealed that the fracture resistance of monolithic translucent zirconia crown is affected by the type of self-adhesive resin cement. The combined application of the MDP-containing zirconia primer with self-adhesive resin cement with different functional acidic monomers, except Panavia SA Cement Plus, influenced the fracture resistance of monolithic translucent zirconia crown. Therefore, the second null hypothesis that the MDP-containing zirconia primer combined with self-adhesive resin cement would not affect the fracture resistance of monolithic zirconia crown, was partially rejected.

Self-adhesive resin cements offer application without the need for additional priming or bonding treatments [24]. However, studies reported that additional priming step improved the bond strength between zirconia and self-adhesive resin cements [19, 30]. MDP primer was chosen because it improves the wettability of the substrate surface for resin bonding and the resulting physico-mechanical interaction within the adhesive interface results in reliable adhesion and favorable chemical stability, supporting the establishment of a water-resistant chemical bond with zirconia [29]. MDP consists of a terminal functional group with phosphoric acid that interacts with zirconia and develops P-O-Zr bonds. The other end of MDP presents C = C bond in vinyl terminal group which enables copolymerization with the resin cement [44].

Air-borne particle abrasion with alumina particles was used in the present study to increase the surface roughness, wettability, and surface energy of the zirconia crown [13, 17]. Also, it was reported that air-borne particle abrasion with alumina particles create hydroxyl groups on zirconia surface which will aid in bonding with both primer and self-adhesive resin cements [7]. Airborne-particle abrasion in combination with phosphate monomer-based primer or resin cement resulted in a long-lasting resin-zirconia bonding [10]. However, air-borne abrasion can produce surface damage, micro-cracks and compromise the mechanical behavior of yttrium-tetragonal zirconia polycrystalline ceramic crowns [6, 15].

The specimens were subjected to thermomechanical aging to simulate approximately one year of clinical service [1]. Thermocycling could alter the properties of resin materials because the absorbed water acts as a plasticizer causing unsupported areas beneath the crown and increasing the risk of fracture under stresses [33, 36]. Also, the water sorption process account for persistent hydrophilicity triggering hygroscopic expansion. It is strongly correlated to the longevity of bonded crowns [8]. The hygroscopic expansion stresses caused by restorative and luting materials were responsible for crack formation in ceramic crowns [24, 37].

The results of the present study revealed inferior fracture resistance with zirconia crowns cemented by Calibra Universal self-adhesive resin cement. These results could be attributed to the high viscosity of PENTA, in Calibra Universal, which may be an issue when the cement paste approaching the surface of zirconia to establish the chemical bonding [25, 34]. Higher viscosity causes ineffective wetting of the entire bonding surface as well as altered micromechanical interpenetration behavior [7, 15]. This in turn have an impact on the restorative system due to uneven dissipation of occlusal loads throughout the entire surface of the crowns [45]. Additionally, it was found that the binding energy of PENTA and its chemical affinity for hydroxyapatite was lower than that of MDP [26]. The use of a “monoblock strategy” for bonding is thought to have boosted fracture strength by allowing the cement to behave as an elastic stress absorber while compensating for the stiffness of the restorative materials [46]. There were no significant differences were found between RelyX U200, Panavia SA Cement Plus, Multilink Speed self-adhesive resin cement groups. Małysa et al. [11] reported that there was a non-significant difference in bond strength between Panavia SA and RelyX U200 regardless of the ceramic type. Regarding Samran et al. [15] the type of self-adhesive resin cement used (Panavia SA Cement Plus, RelyX, SpeedCem) had no effect on the tensile bond strength to zirconia ceramics. Regarding the impact of bond strength on fracture load, these findings may explain the non-significance of the mean fracture load between RelyX U200, Panavia SA Cement Plus, Multilink Speed self-adhesive resin cement groups.

Addition of MDP-containing primer in combination with the studied self-adhesive resin cements, except Panavia SA Cement Plus, significantly improved the fracture resistance of monolithic zirconia crown. The application of zirconia primer increased the bond strength of self-adhesive resin cement to zirconia because of the synergic effect of the MDP and acidic functional monomers [9, 19, 28]. The presence of long carbonyl chains in the MDP structure makes it possible to develop a water-resistant chemical bonding with zirconia [29]. It was found that MDP-containing primer to zirconia ceramics enhanced its bonding quality, but the bond strength of MDP-containing self-adhesive resin cement was not affected by the use of zirconia primer [12, 30]. Yoshida et al. [20] found that an excess of MDP can influence the reactivity of MDP-containing primer and enhance the bond strength of resin cement to zirconia. Chemical reaction between the phosphate group in MDP monomer and the zirconium dioxide on the zirconia surface can be hindered by these groups reacting with other components in ceramic primers. Surface bonding of MDP with zirconia can be inhibited by the phosphate group reacting with other components present within the primer. Hence, excessive Functional monomers such as MDP interfere with the polymerization efficiency of adhesives.

Fractographic analysis showed that the main origin of the fracture was detected at the occlusal surface from the main contact loading area which has the highest tensile stress level. While hackle lines indicated the path or the direction of crack propagation, which propagated corono-apically in all fractured specimens. Burke’s classification was used to identify and categorize the various fracture modes of fractured specimens [47]. When compared to groups with MDP-containing zirconia primer combination, groups without MDP-containing zirconia primer showed a higher rate of catastrophic fracture mode. However, previous study hypothesized that a catastrophic failure mode could result from high fracture load values [48]. This was not the case in the current study, since groups without an MDP-containing zirconia primer had lower fracture load values than combination groups with an MDP-containing zirconia primer.

Increased ceramic resistance to debonding could be observed by fracture that occurred at greater stresses [49]. This might be explained by the restoration’s resistance to debonding at high stress conditions. SEM analysis of combination groups with an MDP-containing zirconia primer has revealed forking “multiple cracks with high stresses” beneath the principal origin. Also, numerous cracks within the cement layer and debonded cement are further signs of excessive stress. With MDP-containing zirconia primer combination groups, debonding expressed cement retained at the shattered zirconia crown rather than tooth structure which could be beneficial to save the underlying tooth structure. This could be explained by the energy of the fracture force being more effectively directed towards the resilient adhesive layer than the tooth structure itself. Consequently, the load accumulated at the crown/cement interface so, the force tends to be dissipated differently than the groups without an MDP-containing zirconia primer.

As limitations, only one type of zirconia crown was studied in this study. Further studies of various zirconia formulations are required. Only axial load is applied in the present study, which does not reveal clinically occurring lateral forces. Also, further studies are needed to compare the performance of self-adhesive resin cements with other types of resin cements. Long-term clinical studies are needed to evaluate the clinical outcomes of zirconia crowns bonded using self-adhesive resin cements.

## Conclusions

Within the limitations of this in-vitro study, it was concluded that;

1. The self-adhesive resin cement with different acidic functional monomer affects the fracture resistance of monolithic zirconia crown.
2. Application of MDP-containing primer could improve the fracture resistance of monolithic zirconia crown with most self-adhesive cements.
3. The application of an MDP-containing primer had no impact on the fracture resistance of monolithic translucent zirconia crown bonded by MDP-containing self-adhesive resin cement.

## Data Availability

The datasets used and/or analyzed during the current study available from the corresponding author on reasonable request.

## References

[1] Kasem AT, Elsherbiny AA, Abo-Madina M, Tribst JPM, Al-Zordk W. Effect of different designs of minimally invasive cantilever resin-bonded fixed dental prostheses replacing mandibular premolar: long-term fracture load and 3D finite element analysis. J Prosthodont. 2022 Dec;10. 10.1111/jopr.13626. 10.1111/jopr.13626 36502276

[2] Stawarczyk B, Keul C, Eichberger M, Figge D, Edelhoff D, Lümkemann N (2017). Three generations of zirconia: from veneered to monolithic. Part I. Quintessence Int.

[3] Camposilvan E, Leone R, Gremillard L, Sorrentino R, Zarone F, Ferrari M, Chevalier J (2018). Aging resistance, mechanical properties and translucency of different yttria-stabilized zirconia ceramics for monolithic dental crown applications. Dent Mater.

[4] Zhang Y, Lawn BR (2018). Novel zirconia materials in Dentistry. J Dent Res.

[5] Zhang F, Reveron H, Spies BC, Van Meerbeek B, Chevalier J (2019). Trade-off between fracture resistance and translucency of zirconia and lithium-disilicate glass ceramics for monolithic restorations. Acta Biomater.

[6] Lawson NC, Jurado CA, Huang CT, Morris GP, Burgess JO, Liu PR, Kinderknecht KE, Lin CP, Givan DA (2019). Effect of surface treatment and cement on fracture load of traditional zirconia (3Y), translucent zirconia (5Y), and lithium disilicate crowns. J Prosthodont.

[7] Comino-Garayoa R, Peláez J, Tobar C, Rodríguez V, Suárez MJ (2021). Adhesion to zirconia: a systematic review of surface pretreatments and resin cements. Mater (Basel).

[8] Kirsten M, Matta RE, Belli R, Lohbauer U, Wichmann M, Petschelt A, Zorzin J (2018). Hygroscopic expansion of self-adhesive resin cements and the integrity of all-ceramic crowns. Dent Mater.

[9] Yi YA, Ahn JS, Park YJ, Jun SH, Lee IB, Cho BH, Son HH, Seo DG. The effect of sandblasting and different primers on shear bond strength between yttria-tetragonal zirconia polycrystal ceramic and a self-adhesive resin cement. Oper Dent. 2015 Jan-Feb;40(1):63–71. 10.2341/13-149-L. 10.2341/13-149-L 25084110

[10] Alammar A, Blatz MB (2022). The resin bond to high-translucent zirconia-A systematic review. J Esthet Restor Dent.

[11] Małysa A, Weżgowiec J, Danel D, Boening K, Walczak K, Więckiewicz M (2020). Bond strength of modern self-adhesive resin cements to human dentin and different CAD/CAM ceramics. Acta Bioeng Biomech.

[12] Stefani A, Brito RB, Kina S, Andrade OS, Ambrosano GM, Carvalho AA, Giannini M (2016). Bond strength of resin cements to zirconia ceramic using adhesive primers. J Prosthodont.

[13] Llerena-Icochea AE, Costa RM, Borges A, Bombonatti J, Furuse AY. Bonding polycrystalline zirconia with 10-MDP-containing adhesives. Oper Dent 2017 May/Jun;42(3):335–41. 10.2341/16-156-L. 10.2341/16-156-L 28467265

[14] Almeida CM, Meereis CTW, Leal FB, Ogliari AO, Piva E, Ogliari FA (2018). Evaluation of long-term bond strength and selected properties of self-adhesive resin cements. Braz Oral Res.

[15] Samran A, Al-Ammari A, El Bahra S, Halboub E, Wille S, Kern M (2019). Bond strength durability of self-adhesive resin cements to zirconia ceramic: an in vitro study. J Prosthet Dent.

[16] Yang L, Chen B, Meng H, Zhang H, He F, Xie H, Chen C (2020). Bond durability when applying phosphate ester monomer-containing primers vs. self-adhesive resin cements to zirconia: evaluation after different aging conditions. J Prosthodont Res.

[17] Xiong Y, Zhao P, Jin C, Wang J, Arola D, Gao S (2021). ï»¿Effect of ï»¿airborne-particle abrasion protocols and MDP-based primer on the bond strength of highly translucent zirconia. J Adhes Dent.

[18] Wiedenmann F, Becker F, Eichberger M, Stawarczyk B (2021). Measuring the polymerization stress of self-adhesive resin composite cements by crack propagation. Clin Oral Investig.

[19] Steiner R, Heiss-Kisielewsky I, Schwarz V, Schnabl D, Dumfahrt H, Laimer J, Steinmassl O, Steinmassl PA (2020). Zirconia primers improve the shear bond strength of dental zirconia. J Prosthodont.

[20] Yoshida K (2021). Effect of 10-Methacryloyloxydecyl dihydrogen phosphate concentrations in primers on bonding resin cements to zirconia. J Prosthodont.

[21] Radovic I, Monticelli F, Goracci C, Vulicevic ZR, Ferrari M (2008). Self-adhesive resin cements: a literature review. J Adhes Dent.

[22] Manso AP, Carvalho RM (2017). Dental cements for luting and bonding restorations: self-adhesive resin cements. Dent Clin North Am.

[23] Ferracane JL, Stansbury JW, Burke FJ (2011). Self-adhesive resin cements - chemistry, properties and clinical considerations. J Oral Rehabil.

[24] Roedel L, Bednarzig V, Belli R, Petschelt A, Lohbauer U, Zorzin J (2017). Self-adhesive resin cements: pH-neutralization, hydrophilicity, and hygroscopic expansion stress. Clin Oral Investig.

[25] Chen Y, Tay FR, Lu Z, Chen C, Qian M, Zhang H, Tian F, Xie H (2016). Dipentaerythritol penta-acrylate phosphate - an alternative phosphate ester monomer for bonding of methacrylates to zirconia. Sci Rep.

[26] Fehrenbach J, Isolan CP, Münchow EA (2021). Is the presence of 10-MDP associated to higher bonding performance for self-etching adhesive systems? A meta-analysis of in vitro studies. Dent Mater.

[27] Madruga FC, Ogliari FA, Ramos TS, Bueno M, Moraes RR (2013). Calcium hydroxide, pH-neutralization and formulation of model self-adhesive resin cements. Dent Mater.

[28] Chuang SF, Kang LL, Liu YC, Lin JC, Wang CC, Chen HM, Tai CK (2017). Effects of silane- and MDP-based primers application orders on zirconia-resin adhesion-A ToF-SIMS study. Dent Mater.

[29] Khanlar LN, Abdou A, Takagaki T, Mori S, Ikeda M, Nikaido T, Zandinejad A, Tagami J (2022). The effects of different silicatization and silanization protocols on the bond durability of resin cements to new high-translucent zirconia. Clin Oral Investig.

[30] Go EJ, Shin Y, Park JW. Evaluation of the microshear bond strength of MDP-containing and non-MDP-containing self-adhesive resin cement on zirconia restoration. Oper Dent 2019 Jul/Aug;44(4):379–85. 10.2341/18-132-L. 10.2341/18-132-L 31216246

[31] Campos F, Valandro LF, Feitosa SA, Kleverlaan CJ, Feilzer AJ, de Jager N, Bottino MA. Adhesive cementation promotes higher fatigue resistance to zirconia crowns. Oper Dent 2017 Mar/Apr;42(2):215–24. 10.2341/16-002-L. 10.2341/16-002-L 27892840

[32] Machry RV, Borges ALS, Pereira GKR, Kleverlaan CJ, Venturini AB, Valandro LF (2021). Influence of the foundation substrate on the fatigue behavior of bonded glass, zirconia polycrystals, and polymer infiltrated ceramic simplified CAD-CAM restorations. J Mech Behav Biomed Mater.

[33] Malysa A, Wezgowiec J, Grzebieluch W, Danel DP, Wieckiewicz M (2022). Effect of thermocycling on the bond strength of self-adhesive resin cements used for luting CAD/CAM ceramics to human dentin. Int J Mol Sci.

[34] Han F, Jin X, Yuan X, Bai Z, Wang Q, Xie H (2022). Interactions of two phosphate ester monomers with hydroxyapatite and collagen fibers and their contributions to dentine bond performance. J Dent.

[35] Shahrbaf S, van Noort R, Mirzakouchaki B, Ghassemieh E, Martin N (2014). Fracture strength of machined ceramic crowns as a function of tooth preparation design and the elastic modulus of the cement. Dent Mater.

[36] Cadore-Rodrigues AC, Machado PS, Oliveira JS, Jahn SL, Callegari GL, Dorneles LS, Burgo TAL, Rippe MP, Rocha Pereira GK, Valandro LF (2020). Fatigue performance of fully-stabilized zirconia polycrystals monolithic restorations: the effects of surface treatments at the bonding surface. J Mech Behav Biomed Mater.

[37] Ms T (2017). Evaluation of compressive strength and sorption/solubility of four luting cements. J Dent Biomater.

[38] Cadore-Rodrigues AC, Machado PS, Oliveira JS, Jahn SL, Dorneles LS, Rippe MP, Pereira GKR, Valandro LF (2021). Surface treatments and its effects on the fatigue behavior of a 5% mol yttria partially stabilized zirconia material. J Mech Behav Biomed Mater.

[39] D’Addazio G, Santilli M, Rollo ML, Cardelli P, Rexhepi I, Murmura G (2020). Fracture resistance of zirconia-reinforced lithium silicate ceramic crowns cemented with conventional or adhesive systems: an in vitro study. Mater (Basel).

[40] Shams A, Sakrana AA, Abo El-Farag SA, Özcan M (2022). Assessment of biomechanical behavior of endodontically treated premolar teeth restored with novel endocrown system. Eur J Prosthodont Restor Dent.

[41] Linhares LA, Pottmaier LF, Lopes GC. Fracture resistance of veneers in premolars. Eur J Dent. 2018 Apr-Jun;12(2):191–8. 10.4103/ejd.ejd_349_17. 10.4103/ejd.ejd_349_17 PMC6004806 29988214

[42] Su N, Yue L, Liao Y, Liu W, Zhang H, Li X, Wang H, Shen J (2015). The effect of various sandblasting conditions on surface changes of dental zirconia and shear bond strength between zirconia core and indirect composite resin. J Adv Prosthodont.

[43] Sakrana AA, Al-Zordk W, El-Sebaey H, Elsherbini A, Özcan M (2021). Does preheating resin cements affect fracture resistance of lithium disilicate and zirconia restorations?. Mater (Basel).

[44] Khanlar LN, Takagaki T, Abdou A, Inokoshi M, Ikeda M, Takahashi A, Yoshihara K, Nagaoka N, Nikaido T, Blatz MB, Tagami J (2022). Effect of air-particle abrasion protocol and primer on the topography and bond strength of a high-translucent zirconia ceramic. J Prosthodont.

[45] Sorrentino R, Triulzio C, Tricarico MG, Bonadeo G, Gherlone EF, Ferrari M (2016). In vitro analysis of the fracture resistance of CAD-CAM monolithic zirconia molar crowns with different occlusal thickness. J Mech Behav Biomed Mater.

[46] Indergård JA, Skjold A, Schriwer C, Øilo M (2021). Effect of cementation techniques on fracture load of monolithic zirconia crowns. Biomater Investig Dent.

[47] Burke FJ, Fleming GJ, Nathanson D, Marquis PM. Are adhesive technologies needed to support ceramics? An assessment of the current evidence. J Adhes Dent. 2002 Spring;4(1):7–22. 12071631

[48] El Shahawy OI, Azab MM. Fracture resistance of prefabricated versus custom-made zirconia crowns after thermo-mechanical aging: an in-vitro study. BMC Oral Health 2022 9;22(1):587. 10.1186/s12903-022-02628-x. 10.1186/s12903-022-02628-x PMC9733029 36494637

[49] Al-Dwairi ZN, Al-Aghbari L, Al-Haj Husain N, Özcan M (2023). Durability of cantilever inlay-retained fixed dental prosthesis fabricated from multilayered zirconia ceramics with different designs. J Mech Behav Biomed Mater.

